# Is it transformation or reform? The lived experiences of African women doctoral students in STEM disciplines in South African universities

**DOI:** 10.1007/s10734-022-00918-5

**Published:** 2022-09-14

**Authors:** Zamambo Mkhize

**Affiliations:** Engineering Mall, University of Cape Town, Room 4.6. Harry Oppenheimer BuildingUpper Campus, Western Cape, 7001 Cape Town, South Africa

**Keywords:** Transformation, Reform, STEM, Intersectionality, Higher education

## Abstract

Science, Technology, Engineering and Mathematics (STEM) fields have historically been disciplines dominated by white men. The colonial ideology designated Africans as subhuman, inferior intellectually, socially, and culturally to the white masculine norm in STEM disciplines. STEM education and careers were thus constructed to attract white, heterosexual, middle-to-upper class, Christian, able-bodied men. This positioning ensured that STEM environments remained inhospitable to anyone whose identity was outside the constructed somatic norm. The calls and imperatives to transform notwithstanding, the transformation process in STEM disciplines is moving at a snail-like pace. This article argues that what is occurring in STEM disciplines in South African universities is reform not transformation. It is underpinned by the intersectional theory within the qualitative paradigm. Seventy-three African doctoral and postdoctoral women students in STEM were interviewed from five South African universities. The findings highlighted how African women in STEM face challenges based on their racial and gendered identities and that what is presented as transformation is still oppressive to them. The study also found that equity through access to education in democratic South Africa does not equate to transformation. The argument presented is that despite existing policies and initiatives in South African universities to transform, the demographic inclusion of African, female staff and students does not necessarily equate to transforming the STEM environment. What needs to occur is a shift beyond reform and towards transformation through the use of strategic inventions which dismantle the racist, sexist, classist, and xenophobic ideology that permeates these environments.

## Introduction

STEM fields have historically been disciplines dominated by white men. According to Braselmann (2003), Borum and Walker (2012), and Herzig (2004), because of the ideology of western patriarchy that believed that the female gender is intellectually inferior to the male gender, women were not deemed intellectually suitable to pursue STEM disciplines. The science community created a racial hierarchy amongst people to justify racial and perceived differences, especially around the intellectual and social inferiority of African people (Mills, 1997; Wilkins-Yel et al., 2019). This shaped the constructed and productive knowledge in the science community, institutionalizing racism, and sexism. The construction of science and race are linked to how academic talent in the STEM disciplines is grounded in racialised and gendered notions of superiority and inferiority. STEM in South Africa has a history of exclusion. During apartheid, there were structural and cultural restraints imposed on women, hence why white males were the overwhelming majority enrolled in science and engineering higher education courses (Case & Jawitz, 2004; Cruise, 2011; Martineau, 1997; Moshupi, 2013). In contrast, African people required special permission to attend the white universities offering STEM courses or were forced to pursue these particular degrees abroad (Mlambo, 2017).

Changes to the STEM environment have proceeded at a glacial pace and racist and sexist ideologies remain in place in these disciplines. Despite the call for transformation at universities being heeded, according to Mkhize and Idahosa, (2021), STEM disciplines and environments remain untransformed. Instead, what has been occurring is reform because there are large numbers of African students presently in STEM disciplines. STEM education and careers were designed to attract white, heterosexual, middle-to-upper class, Christian, able-bodied men. This highlights how the ontology and epistemology are co-constructed in STEM (Herzig, 2004; Mkhize & Idahosa, 2021). The ontology of STEM being a white domain co-constructs the belief that innateness of STEM abilities is solely for white people or males. Studies (Battey & Leyva, 2016; Jett, 2022; Leslie et al., 2015; Leyva, 2017) have been conducted in the global north which found that women and Black people are underrepresented in STEM fields because of the belief that raw, innate talent is the main requirement for success in all STEM fields; therefore, women and Black people are stereotyped as not possessing such talent. This is why the STEM environment is inhospitable to anyone whose identity does not fit any of those categories. Universities in South Africa have been actively trying to transform the STEM disciplines by enacting policies which are targeted towards attracting African students (Badat & Sayed, 2014; Nyamnjoh, 2016), especially African women to these scarce skills fields, yet they remain untransformed. Affirmative action was met with resistance by the dominant white group in these fields and yet, the biggest beneficiaries of affirmative action have been white women (Borum & Walker, 2012; Mugambwa, Mwebaza & Namubiru, 2017). White women have enormously benefited from affirmative action policies in STEM (Borum & Walker, 2012; Collins et al., 2020; Mugambwa, Mwebaza & Namubiru, 2017), and consequently, most of the literature on issues of exclusion and marginalisation in STEM is based on white women’s experiences (Borum & Walker, 2012; Collins et al., 2020). As a result, STEM disciplines believe they are transforming the system by the addition of white women. However, African women remain extremely underrepresented. This miniscule number of African women has consequential implications to their contribution and representation in the STEM fields.

This paper aims to contribute and extend the debates around transformation by focusing on what is occurring in STEM disciplines in South African universities, which is reform and not transformation. As Essop (2020) reiterates, reform pertains to reforming a system, in this case giving access to people who had previously been denied due to apartheid legislation. Transformation, on the other hand, means fundamental change, change in the institution, in STEM departments, and changes in ideology of who is a legitimate knowledge bearer within the STEM disciplines. In Mkhize and Idahosa (2021), they attest that although there has been a high female enrolment in STEM disciplines, they have been at undergraduate levels, and men continue to be the majority in postgraduate levels (DWYPD + 25 SA Country Report 2019). In South Africa, Black women continue to be underrepresented compared to their male counterparts, particularly at postgraduate levels (DWYPD 2020). In 2018, of the total number of African women enrolled in STEM disciplines, 74.3% were in undergraduate levels, and the number declined the higher the degree to 15.07% at postgraduate diploma and honours levels, 8.16% at masters level, and 2.25% at doctoral levels (HEMIS 2018). Within the period 2008–2018, African women made up 33.9% in 2018 and 27.5% in 2008 of the number of enrolments in STEM disciplines and 10% in 2018 and 7.7% of the total number of enrolment respectively. Graduation rates reveal similar patterns of inequality. Despite the high graduation rate at undergraduate levels with women representing 62.5% of the graduates in STEM disciplines, of this percentage only 10% graduated at masters level and 2.1% at doctoral levels in 2018 (HEMIS 2018). The statistics reveal that women in STEM lag behind their male counterparts in terms of doctoral attainment, reifying Ong (2010), assertion that most women in STEM are white and most Blacks are male (Idahosa & Mkhize, 2021). There are a promisingly large number of African students entering the STEM disciplines but for a number of reasons they do not progress to the doctoral level of these disciplines or become academics. These reasons are epistemological, ontological, institutional, and socio-cultural factors, and the hostile environments of STEM, as well as personal career choices. According to Mkhize and Idahosa (2021) and Mlambo (2017), the entire STEM field needs to be transformed but higher education institutions continue to be plagued by racism, sexism, racialised bullying, sexual harassment, and stereotyping which is a deterrent to African women entering and remaining in STEM academia. By observing the foundational structures of STEM, by looking at how racism operates in the experiences, ideologies, practices, and policies of STEM training programmes, I argue in this paper that this could lead to true transformation in STEM disciplines, instead of what has occurred, which is reform of the system masquerading as transformation.

The #ShutDownSTEM was a global protest which took place in June 2020, which saw thousands of Black scientists protesting against racism in science and academia. It highlighted how globally untransformed the STEM industry and universities remained. Globally, Black scientists were angry and tired of the lack of transformation in STEM and the racism they continued to endure in the field and in academia. In a time during a pandemic where STEM fields are in sharp focus, as the leaders of people experimenting and finding cures to urgent viruses and diseases, STEM remains plagued by its racist, sexist, colonial, and imperial disease. The Black scientists wanted substantial change and were no longer interested in seeing scientific organisations and universities releasing statements condemning racism and pledging promises but having no effective action plan to combat it. The scientists had specific suggestions to transform STEM, such as having more Black science academics write review articles and peer review scientific papers and serve on STEM editorial boards. The Black scientists saw the protests of 10 June 2020 as the beginning of a process because no one race has a monopoly on intelligence, creativity, and ideas in STEM (Chen, 2020; Grant, 2020; School of Science MIT, 2020); ; ; ; . In South Africa, STEM disciplines remain overwhelmingly white and yet African people comprise the majority of the population.

Most of the literature around reform in higher education in South Africa is centred around curriculum reform (Council on Higher Education, 2013; Elliott, 2005; Grobbelaar, 2004; Heleta, 2016; Lumadi, 2021; Mama, 2003; Oluwakayode, 2021; Shay et al., 2016). Research has been conducted on reform in STEM (Gaotlhobogwe, 2019; Mlambo, 2021) and transformation of STEM in South Africa (Babalola et al. 2021; Idahosa & Mkhize, 2021; Liccardo & Bradbury, 2017; Liccardo et al., 2015), but research on reform being concealed as transformation in STEM fields is scarce and that is where the novelty of this study lies. This article begins by briefly outlining the literature of African women in STEM in South African higher education and the use of intersectionality theory, followed by the research methods and thereafter the findings, discussion, and conclusion.

## Background

### Reform, transformation, and African women in STEM

#### Historical legacies of inequality in higher education in Africa

It is important to foreground that the colonisers never designed higher education for Africans (Heleta, 2016; Minga, 2021; Mohamedbhai, 2014). However, Nhemachena et al. (2020) showed that universities existed in pre-colonial Africa where subjects such as mathematics, science, and human anatomy were taught to African students. In African states, social institutions of higher learning are still mostly being organized according to the parameters of the colonial legacies with regard to the nature of the institutions, and the criteria utilized in affording access to them (Assié-Lumumba, 2006, p. 9). Education was expected to be a powerful instrument for redressing the structural inequality embedded in society (Department of Higher Education & Training White Paper, 1995); conversely, the roots and contemporary challenges of the structural gender imbalance remain. Mama (2003) argues that after the establishment of independence, African universities did not make the shift away from the colonial modes of organisation and intellectual life. African universities remain ‘the male preserves, dedicated to the production of good colonial subject’ (Mama, 2003, p. 105). Mama (2003) reiterated that what persists in these institutions are institutional and intellectual cultures of the colonisers which were permeated with sexual and gender dynamics, which make higher education environments hostile for women (Mama, 2003, p. 118).

#### Reform in higher education

Post-democracy, universities in South Africa have had three challenges, to reproduce and retain the next generation of academics, transform the historical social composition of the academic workforce through equity and redress, specifically for African females, while transforming, retaining, and enhancing the academic capabilities of the next generation, using intellectual and academic capabilities related to teaching, learning, research, and community engagement. Nonetheless, what is presented as transformation in academia is reform. According to Nodoba (2020), reform revolves around strategic moves within current structures and systems. South Africa after the 1994 democratic election opened access to Africans to institutions and spaces they had previously been denied. This emancipation of access was the beginning of reform. Transformation, on the other hand, means fundamental change, change in institutional structures and systems and change in social justice in higher education. Many people believe that since there is reform there is transformation and that is inaccurate. Reform pertains to reforming a system, in this case giving access to people who had previously been denied, due to the apartheid legislation. Transformation, on the other hand, entails moving beyond just reforming the system towards allowing those previously denied sincere representation in the upper echelons of power in higher education institutions, as well as refiguring the system towards social justice for all people. The effects of democracy are such that universities remain a microcosm of society, which is still untransformed. In universities, transformation and liberal ideology are connected. Neoliberalism and neo-colonialism exist in the academic world and the norm is whiteness (Nhlapo et al., 2020; Settles et al., 2019). Neoliberalism has been the dominant ideology in universities since the beginning of the twenty-first century (Boughey & McKenna, 2021). Neoliberal policies demand all students be the same, come from the same educational backgrounds, and to be able to enter and leave universities in a timely manner. This ‘sameness’ demanded by neoliberalism leads to universities promoting meritocracy. This meritocracy further disadvantages African students because most come from poor rural and urban areas where they are taught subjects in their indigenous language and are not fluent in the language of English. The participants come from disadvantaged secondary schools and need to learn many things before they enter university. In recent years, due to the encroaching neoliberal policies and the commodification of knowledge combined with decreased government subsidies, universities have been forced to use the neoliberal business model and fund their own institutions. If African students or African staff challenge these ideologies and policies, white fragility exposes itself and that results in the resisters being labelled as ‘racists’ for wanting to dismantle a system that was never designed to educate or advance the African indigenous people of South Africa (Msibi, 2020; Nhlapo et al., 2020). Reform and neoliberalism are closely connected because reform is a project of neoliberalism. Transformation, on the other hand, requires a far more nuanced and extensive change than neoliberalism entrails.

Reform exposes the conundrum that having African representation does not change anything because the problems that plague African students are systemic; the viable solution is a complete overhaul of the system. According to Mboti (2021, p. 49), apartheid was colonisation on steroids. It was and is never the same thing. It changes, mutates, and adapts to hide itself better. Reform can be disguised as transformation when it is not transformation, to distract the critics who want to dismantle the system when all the system wants to do is to remain and metamorphose without relinquishing any power. A mass number of African students does not equate to transformation, but a change in structural configuration is necessary for real transformation to occur. Reform can either lead to transformation or catalyse to a backlash as previous student protest movements such as #RhodesMustFall and #FeesMustFall have shown us.

Reform works around strategic moves and strategic appointments, whilst the ethos of the system remains in place. Ndoda (2020) argues if there is transformation why is there a need for decolonization. Instead, what has occurred has been the modification of the same structure, which was never designed to educate or advance the African nation but was there to ensure the perpetual servitude of the African people (Mboti, 2021; Msibi, 2020; Ndoda, 2020). STEM disciplines proclaim to be neutral and objective but in truth, they are masculine and white-centred (Mkhize & Idahosa, 2021). The question becomes how STEM disciplines, which historically have worked to prove racial and gender superiority over the Africans, can truly transform to a more equitable and inclusive in their environment.

#### Racial transformation in higher education

The democratic era of 1994 resulted in the sustaining of white imperialism and imperial thought and African people were accommodated as long as they did not threaten the white people’s comfort (Msibi, 2020). In the post-democracy era, South Africa had ‘*Good Intentions*’ transformation, which Msibi (2020) argues focused on the inclusion of African people in white spaces rather than true transformation and change. True transformation, as Msibi (2020) and Ndoda (2020) argue, is transformation of equity, fairness, and social cohesion in institutions that promoted violence and exclusion of African people, such as white universities. White institutions were a project of colonisation, with the sole agenda of ‘divide and conquer the natives’ (Nhemachena et al., 2020, p. 33). Universities foster and support white notions of superiority and excellence and association with whiteness to maintain the status quo.

According to Mboti (2021) and Msibi (2020), the system exists to erase blackness and does not welcome it, or see it, and consequently, Africans must assimilate into the existing system. The systemic structures and barriers African students face are colonial constructions that persist today and show that white power will not relent and will push back constantly (Mboti, 2021). A particularly insidious form of racism that exists within the universities and STEM discipline is the colour-blind ideology. This ideology perpetuates the idea that it is rude to recognize a Black person’s race, ethnicity, or culture. This ideology works in conjunction with the neoliberal ideology of meritocracy along three lines. First, it refuses to acknowledge that racism and sexism are real issues in current higher education. Second, it fails to recognize how institutional inequality continues to bestow unearned advantages to whites. This denial allows the dominant ideology to locate racism today in a few prejudiced individuals. Third, it fits with framing lower achievement by African students as due to their biological and cultural deficiency, which essentially blames African students for their lower status and perpetuates the historically STEM ideology of Africans being inferior intellectually, culturally, socially, and biologically to whites. It also shows an unwillingness to question how institutions continue to benefit whites, coupled with statistics showing lower achievement scores for Africans, and shifts the blame to African students, their families, their communities, and their culture and away from whiteness. The ideology works with tokenism and the academic rhetoric that is protracted is that resilience is the key to success and career achievement in STEM. This untruth dismisses the invisible forms of racism such as structural and institutional racism, which are the impenetrable barriers to African students thriving and succeeding in STEM fields (McGee, 2021, p. 7).

Msibi (2020) argues that historically universities had a mystique. #FeesMustFall cut through that mystique and showed the academic world as a highly violent space for anyone who was not white, male, middle-to-upper class, or heterosexual. The consequences of the #FeesMustFall movement demonstrated that African students were led to believe that transformation was occurring, whereas it was merely reform. According to Mkhize (in press), African students in universities and specifically African women in the STEM disciplines reported that they were either encouraged to reconsider pursuing STEM disciplines or told to be grateful to have been ‘allowed’ to pursue STEM degrees. They were expected to be quiet and submissive and to constantly prove their intellect to show that they were worthy to be in those STEM disciplines.

#### African women in higher education in South Africa

In South Africa, the minority status of African women in higher education is juxtaposed against their numerical majority status in broader society. The positioning of African women in higher education in South Africa as the majority population provides a unique perspective compared to the USA and Europe where African women are minority populations (Mlambo, 2017, p. 40). The low representation of doctoral students needs to be understood in relation to the number of qualified African women capable of occupying academic positions. If the number of African women in doctoral degrees remains low, this directly impacts the number of potential future African women academics in that field. Identifying the reasons why African women in African majority contexts are absent in academia provides insights into the global and cross-cultural nature of African women’s underrepresentation in STEM (Mlambo, 2017, pp. 40–41). Samson et al. (2021) mention pipeline thinking when arguing for way to correct the injustices of the South African past in terms of structural and institutional racism that persists. Samson et al. (2021) discuss how to bring about transformation by attempting to create a straight trajectory for students from undergraduate to postgraduate and eventually enter the academy as academics (p. 1208). Although Samson et al. (2021) focus on funding, managerial policies, and lack of support for postgraduate students, this article focuses on the experiences of doctoral African students in STEM disciplines and what occurs in these disciplines which discourages them from becoming academics. Numerically, African women are graduating at a higher rate with at 63% in all fields in undergraduate degrees, which is almost double that of white females. This illustrates that there are structural and institutional barriers that prevent them from progressing to postgraduate degrees, specifically in STEM (Mkhize & Idahosa, 2021). African women professors, women, and African women remain grossly underrepresented in other senior administrative positions and their standpoints expose them to unique challenges and experiences (Mlambo, 2017, p. 42). African students represent the majority (75.6%) of the student population, with African women being the majority. The Department of Higher Education and Training (2020) indicates that 42.7% of the academic staff are white people, despite white people making up only 9% of the country’s population. African students in this current study reported having never been taught by an African female lecturer or professor while others said they were taught by African lecturers from other African countries who were mostly males, in their undergraduate and postgraduate studies. There are huge gaps pertaining to students’ progression into postgraduate degrees and what is being presented as success is not really a true reflection, when we look closely at the numbers. While African students constituted 84.8% of the enrolments in 2017, compared to 74.5% in 2005, the participation rate for African students increased from 12% in 2005 to 18% in 2017. This is still below the overall participation rate of 21% and far below the participation rate of white (56%) and Indian (47%) students in 2017 (Essop, 2020).

Transformation cannot be understood as a blanket term because it is applied differently in different institutions. Every institution has its own institutional culture and challenges, which require a contextualised approach to change (Ramohai, 2019, p. 2). In the South African context, the institutions of higher education have very different transformation needs. Previously, White Afrikaans^1^ institutions faced more challenges relating to racial inequalities than previously Black institutions. All higher education institutions in South Africa are subject to rules and regulations and one of them is to prioritize African women in the transformation initiative (Badat, 2010 in Ramohai, 2019, p. 6). According to Mlambo (2017), African professors in South African universities make up 4% of professors and 0.85% represents African women professors. There was a 19.9% increase of African professors in 2017 in South African universities and African women now represent 4.2% of the professoriate in 2020 (Higher Education Data Analyzer, 2020). According to the Department of Higher Education (DHET) (2020), African academic staff (African, Coloured, Indian, or Asian) represent only 39.6% of academic staff at public universities. White people represented the largest population proportion of academic staff at 42.7% (DHET, 2020). In a country of almost 60 million, African people are the majority and white people make up less than 9% (Mlambo, 2021, p. 158). Africans are over-represented in support roles where they occupy about 97% of administrative and service positions (Mabokela & Mlambo, 2017). This shows that reform is occurring but not genuine transformation.

## Intersectionality

The analytic approach employed in this study is grounded in the intersectional perspective to examine factors to see if transformation or reform is occurring in STEM disciplines that affect African women in five South African universities. Kimberley Crenshaw (1989) coined the term intersectionality, but it is rooted in Black feminist thought. Intersectionality described the way multiple identities, based on race, gender, class, ethnicity, nationality, and sexuality, are systematically and structurally oppressed. These structures systematically oppress African women in ways that do not persecute African men or white women. African men face racial discrimination but have gender privilege, and white women may experience sexism but have racial privilege, whereas African women face racial and gender oppression in STEM disciplines (Mkhize & Idahosa, 2021). These axes of difference are not merely descriptive, but they co-constitute each other (Slater & Liz, 2018, p. 341). Cho et al., (2013) state that what makes an analysis intersectional is the adoption of an ‘intersectional’ way of thinking about the issues around sameness and difference and their relationship to power.

According to Nash (2008), intersectionality focused on two main areas, the race/gender binary which argues for an understanding of the multi-dimensions which includes ways in which race, gender, class, ethnic identity, and context affect experience. Intersectionality seeks to reveal the ‘racial variations within gender and the gendered variations within race, through its attention to subjects whose identities contest race or gender categorization’ (Nash, 2008, pp. 2–3). This means that by focusing on the experiences of African, working class women, white women, and homosexuals, intersectionality opens up possibility for understanding the ways in which social positions (race, gender, class, ethnicity) shape and influence identity formation (Mkhize & Idahosa, 2021). Intersectionality moves beyond race and gender to consider the ways in which political milieus affect identity and individual experience; for example, colonialism, capitalism, and nationalism are incorporated into intersectional analysis (Levine-Rasky, 2011). Nash (2008, p. 8) averred that this conceptualization allowed the researcher to understand the legacy of exclusion in the South African context on multiple subjects, and in doing so, avoided the problems of essentialism and exclusion of particular groups. Such situatedness allows the author to understand the experiences of African women doctoral students in relation to their historical and social context, as well as understanding whether transformation or reform is occurring in these institutions.

## The study (Table 1)

**Table 1:** Table of participants^a^. All the names are pseudonyms

No.	Name	Discipline	Institution
1.	Zethu	Virology (2)	A
2.	Mpumi	Immunology (3)	A
3.	Mahlohonolo	Clinical and laboratory science	A
4.	Makhosazana	Microbiology (1)	A
5.	Shudufhadzo	Medical science	A
6.	Nompumelelo	Public health	A
7.	Rego	Physiology (2)	A
8.	Nokuphila	Medical science (1)	A
9.	Thembeka	Forensic entomology (2)	A
10.	Winnie	Neuroscience	A
11.	Philisiwe	Medical microbiology (PD)	A
12.	Thandeka	Microbiology (4)	A
13.	Zandile	Mathematics (1)	A
14.	Nelisiwe	Mathematics (2)	A
15.	Nelisiwe	Public health (4) final	A
16.	Sindisiwe	Electric engineering	A
17.	Londiwe	Statistics (1)	A
18.	Ntombi’enhle	Chemical engineering	A
19.	Mbaliyezwe	Astrophysics (3)	A
20.	Relebohile	Chemical engineering	A
21.	Thokozile	Physics (1)	A
22.	Zakifa’intombi	Mathematics	A
23.	Vivian	Mathematics	A
24.	Monica	Mathematics (PD)	A
25.	Duduzile	Bio-statistics	A
26.	Thandekile	Public health (4)	A
27.	Nokukhanya	Medical microbiology (4)	A
28.	Xolisile	Neuro-science	A
29.	Abi	Botanical science (PD:3)	A
30.	Nonceba	Phytopathology and advanced mycology	A
31.	Zenzile	Plant pathology	A
32.	Thembekile	Chemical engineering (5)	Z
33.	Thabile	Biological sciences (1)	Z
34.	Basetsana	Plant biotechnology (1)	Z
35.	Bathobile	Science (PD:3)	Z
36.	Nomthandazo	Oceanography (4)	Z
37.	Nonhlanhla	Oceanography (2)	Z
38.	Lindiwe	Chemical engineering	Z
39.	Ntombi’xolo	Mathematics	X
40.	Thando	Physics (3)	X
41.	Nomvula	Chemistry (2)	X
42.	Zamantshali	Astrophysics (1)	X
43.	Thembani	Plant microbiology (4)	X
44.	Mandisa	Physics (submitted)	X
45.	Thobe	Computer science (2)	X
46.	Leleti	Environmental chemistry	X
47.	Thuliswe	Microbiology	X
48.	Nomvuyo	Microbiology (3)	Xx
49.	Anele	Microbiology (2)	Xx
50.	Khayakazi	Microbiology	Xx
51.	Fezile	Microbiology (1)	Xx
52.	Boniswa	Plant pathology	Xx
53.	Lungi	Microbiology (1)	Xx
54.	Maud	Zoology	Xx
55.	Nomzamo	Pharmaceutical science (1)	B
56.	Philile	Biochemistry and microbiology	B
57.	Sindiswa	Science	B
58.	Doreen	Science	B
59.	Carol	Science	B
60.	Nonku	Science	B
61.	Zintle	Science	B
62.	Zanele	Biology (A)	B
63.	Sonto	Science	B
64.	Nokuthula	Science	B
65.	Alude	Science	B
66.	Thandi	Chemistry and physics	A
67.	Khanyisa	Microbiology	Xx
68.	Enele	Science	X
69.	Nonjabulo	Organic chemistry	X
70.	Amkhita	Biology	X
71.	Thubelihle	Science	X
72.	Aphile	Science	X
73.	Khanyanjalo	Science	B

^a^Code

(Year): Year in PhD.

University Codes

University A: (Historically Black)

University B: (Historically Black)

University Z: (Historically White)

University X: (Historically White)

University Xx: (Historically White-Afrikaner)

This study is situated within a critical research paradigm whose purpose is ‘to identify, contest and help resolve power imbalances’ in society, which contribute to ‘system inequalities and injustice’ (Taylor & Medina, 2013, p. 6). This article employed a qualitative research methodology, where semi-structured interviews were employed after obtaining ethical approval from the universities. Seventy-three participants, five of whom were early career staff and postdoctoral fellows in STEM disciplines, were interviewed in five South African universities. The universities were selected because they ranked in the top five in South Africa in that year, and the researcher wanted to explore what those ranking entails in terms of transformation within STEM disciplines. Two of the institutions are historically Black universities (catered to African students but medium of instruction was English) and the rest are historically white (British origins—English speaking) institutions, with one historically Afrikaans^2^-speaking university.

Given the contextual particularities of all five universities, examining the experiences of African female doctoral students in STEM provides insight into how prevailing institutional conditions enact transformation and implement it. Purposive sampling was employed aimed at identifying African female doctoral students in STEM disciplines at the universities after ethical clearance was obtained from all the institutions. Only students who self-identified as African women, pursing their doctoral degrees in STEM disciplines in these universities, were recruited to be participants in this study. The participants’ ages ranged from 27 to 44 years old and they were in various years in their doctoral degrees. In-person interviews began in 2019 and then shifted to virtual interviews due to the SARS-CoV-2 pandemic. The interviews lasted between 45 min to 1 h. The questions began by asking the participants their demographic information such as name, age, and degree. Then, the questions moved to asking them about their motivations for entering their STEM degree of choice, their experiences in those disciplines, and their thoughts about transformation of STEM in higher education in South Africa. The interview ended by asking participants their understanding of transformation in STEM and their interest in becoming academics. The participants were guaranteed confidentiality and therefore, all the names used in this article are pseudonyms. The interviews were conducted with the intention of understanding the participants’ experiences in STEM disciplines. The interviews explored the participants’ location within the institution and their experiences, which either enabled or limited them, as well as probing into their understanding of whether reform or transformation was occurring in their STEM disciplines.

A thematic analysis of the data was employed using NVivo data analysis software (Bazeley & Richards, 2000) to analyse how participants’ social positions (being woman and African) intersected with the historical and current political milieu, to impact their experiences in STEM disciplines. The data was coded for patterns in the intersectional experience of participants, to understand what the main factors were that impacted their inclusion and retention in STEM disciplines (Saldaña, 2009). Participants cited an intersection of institutional, disciplinary, cultural, and interpersonal factors as responsible for their decision to remain in academia or enter industry after they attained their doctorate degree. The themes that emerged revealed structural, institutional, and disciplinary issues such as socio-economic status, and an intersection of social positions such as race, gender, class, and age with historical legacies, departmental politics, and the complexities of ethnicity. Cultural issues like social norms, gendered stereotypes, and discrimination and interpersonal issues like feeling alienated, isolated, excluded, and lack of mentors impacted their academic journeys. Regarding the interpersonal aspects, the participants noted feelings of not belonging, based on their identities, and they had to constantly prove themselves as intelligent and legitimate knowers within their STEM disciplines.

## Limitations

The study was conducted between the years 2019 and 2021 and only in five of the twenty-six universities in South Africa. The limitation was that the sample size may be small and if all universities in South Africa were incorporated the data may have been different. Some participants were interviewed in particular years in their doctoral studies and perhaps if there was a follow-up their experiences and narratives would have shifted. A study over a period of years can also test causal patterns in predicting certain codes or categories which I was unable to test with my data. Perhaps a longitudinal study in the future may focus on that. It would be interesting to be able to investigate the experiences of African men in STEM disciplines and their perceptions and understanding of transformation and reform in STEM in South African universities.

In the next section, I discuss the findings and examine socio-cultural and interpersonal obstacles, the intricacies of xenophobia, and the ‘transformation’ mutations which will unpack how transformation has ‘altered’ to further oppress the people it was designed to liberate. That section will elucidate whether transformation or reform is occurring in these STEM disciplines for African women in universities.

## Findings and discussion

This section discusses the themes that arose from the study. Consistent with the literature, the study participants cited an intersection of socio-cultural and interpersonal factors, the complexities of xenophobia within African people in the STEM departments, and how ‘transformation’ has been amended to represent something different. The narratives revealed how socio-cultural and interpersonal factors like the participants’ race, gender, and socio-economic status situated them in complex and marginal positions (Mkhize & Idahosa, 2014).

### Socio-cultural and interpersonal obstacles

A finding of this study is that the socio-cultural and interpersonal obstacles African women encounter such as racialised and gendered discrimination highlight how the STEM disciplines remain untransformed. These findings reveal that what has occurred is demographic reform but that racist, sexist, and classist ideologies persist within the STEM disciplines, as narrated by the participants who shared their experiences, as Zamantshali explained,

Men need to be part of the transformation agenda. Men, particularly Black men discriminate against Black women. Black men fear white women because they do not ask them questions such as ‘why are you getting your PhD?’, ‘Who will marry you with a PhD?’, ‘When will you leave and have children?’ As a Black woman you are suffocating, you face discrimination from white men and women and then you are harassed by Black men, it is a lot to deal with.

Zamantshali’s narrative focused on the intersection between socio-cultural norms and gendered stereotypes. In African cultures, African women still face societal gendered expectations, of getting married and bearing children first before striving for a professional career. As another participant, Lindiwe stated African women continue to receive discouraging messages from African men even within academic spaces. Lindiwe further reiterates,

There is a pattern I have noticed in meetings where the Black men are extremely harsh in their critique of Black women’s work and their projects, unnecessarily so. They are unsupportive and only offer negative comments. They are harsher towards us than their white counterparts.

Lindiwe’s narrative highlights the racialised patriarchy African women within STEM experience from African men. As McGee (2021) argues, this public humiliation of African women by African men perhaps stems from the need for African men to assert themselves publicly in front of their white counterparts, since they are unable to do so to the white men and white women because of their racial privilege. Therefore, the African woman is the only remaining target they can assert their gendered privilege. The participants having achieved academic success by pursing their doctoral degrees in difficult STEM disciplines are constantly reminded they are rebelling against the expected norm of the socio-cultural designated identity of being wives and mothers. This shows the insidious way patriarchy works; these messages from African men could at best be viewed as a reminder to women to remember their constructed and expected roles or face being ostracized from their societies. At worst, these messages are deliberately designed by African men to embarrass African women in front of their white peers and to distance themselves from being associated with them and their ‘negative perception of intellectual inferiority’. Or it could be to actively discourage African women from being too ambitious because African men will also have to compete against them and they are already competing against all white people. This oppressive type of behaviour from white, Coloured, and Indian women, as well as African men, emphasizes the intersectional oppression experienced by African women which is different from how oppression is experienced by African men and white women.

Fezile’s narration mentioned the oppression African women consistently face perpetuated by other women within the science disciplines. Fezile said,

White Afrikaner women are horrible they are so racist and treat us as if we are intellectually inferior because we are Black women. Yet these same women believe they are as oppressed as us because they are women, but they themselves oppress Black women worse than their white men do.

Other African women in the study mentioned the problematic white women in STEM, who believe that African women have a common experience in that they too are discriminated against by white men, yet as Fezile (and others) explained there is a sisterarchy at play. Sisterarchy, a term coined by Nzegwu (1990), explains that all women are united by their being oppressed, due to their gender. However, there is also a racial hierarchal system of oppression within that unity, with white women perpetuating oppression onto African women. Thandeka’s and Zamantshali’s narratives illustrate the untransformed institutional culture that permeates STEM disciplines.

Another participant, Thembani, said,

Black students are viewed as labourers, domestic workers and are intellectually undermined. White students are privileged and have more power and get away with a lot of stuff in the lab. The white males are aggressive to Black women, they are physically aggressive, and they shout at Black women in their faces.

As Thembani’s narrative explained, the students who insult, demean, and harass the African women students felt safe in doing so because the STEM departments and institutions did nothing to stop or prevent it. Their attitudes and inaction had implicit departmental and institutional support. Thus, it is not surprising that the neutrality, colour-blind, meritocratic, and toxic levels of prejudice remain unchallenged in STEM disciplines, because other races have the implicit protection of the institution and STEM departments that allows them to badger African women unchecked as Thembani’s experience highlighted. That once again highlights how reform only is happening in these STEM spaces, because if the STEM disciplines were truly transformed, none of those students would feel emboldened enough to continue repressing African women. As Thandeka mentions,

Black female students face many hardships which are deliberately caused by the Indian women in the departments. Black women must be strong and preserve facing many obstacles.

Thandeka’s narrative emphasizes the experiences of African women who face oppression from other women classified as Black too (Indian and Coloured^3^). Intersectionality theorizes around this issue of within-group discrimination and of colourism and hierarchies that persist within the black community (of Black, Indian, and Coloured) which historically was perpetuated and manipulated by the apartheid government for their own agenda of sowing division amongst the races. Thandeka and the other participants spoke about the racism they experienced from other women but did not engage with the sexism. Since the oppression they experience is perpetrated by other Black women, race cannot be the only factor, but they do not engage further with this type of oppression being sexist in nature. Sexism, however, is usually theorised as something that men do to women and that is simplistic. What we need to interrogate is that oppression inflicted on women by other women is more nuanced than just being about race or ethnicity. Perhaps what is needed is a discussion around the patriarchy and misogyny that exist in the STEM disciplines and not the one perpetuated by only men; but that is beyond the scope of this paper.

The issues raised in this theme accentuate how untransformed the STEM disciplines remain. A truly transformed environment would actively encourage still disadvantaged^4^ people to remain, succeed, and attain their doctorate degrees. Many participants in this study mentioned that the first reason they chose to pursue their doctorate in STEM disciplines was because they wanted to be researchers and professors and to contribute to their societies, as well as to be the role models and mentors they did not have as students. Entering the industry, although more fiscally lucrative than academia, was a secondary option. However, if STEM environments continue to perpetuate their historical racial and gender hierarchal discrimination, transformation will not occur because African women will opt to go into industry instead of academia for those reasons and what will continue to happen is reform by demographical numbers in these fields but not transformation.

### Complexities of xenophobia

During colonialisation and later apartheid, Africans were segregated according to their ethnicities. African migrants had been coming to South Africa during the colonial and, later, in the apartheid times. On the other hand, after 1994, they began to enter South Africa in large numbers driven by the political and economic hardships in their own countries. The media portrayed African foreigners negatively and they were blamed for the ills of the country. Matsinhe (2011) argued that South Africa positions itself as the superior power on the continent and Africans from other African countries are portrayed as inferior and an ‘us’ versus ‘them’ mentality persists. In South African universities, there is a push for transformation and that entails hiring and retaining indigenous Africans. This agenda has caused tensions and competition amongst indigenous Africans and non-indigenous Africans, due to the limited resources and academic positions available. Xenophobic attacks in the media have been presented as something perpetrated by indigenous Africans on non-indigenous Africans. However, my data suggests that in STEM disciplines in South Africa, the opposite is often occurring as Nompumelelo narrates,

There is overt xenophobia in my university and in my discipline and it is the African foreigners against the Black South African students. There is this consistent narrative that Black South African students are slackers and do not deserve all the good things their government gives them, such as scholarships and funding for their research. African foreigners work harder and faster and are more intelligent. There is this one African foreigner professor who is known for deliberately sabotaging Black South African students by not helping them to progress, so they get frustrated and do not complete their PhDs. Yet the African foreigner students he supervises always graduate in record time and it feeds into the narrative that this professor perpetuates that Black South African students are unintelligent and do not graduate. The same professors treat Indian and white women PhD students very well, unlike the way they treat Black women students.

Xolisile also mentioned the xenophobia that exists in her institution as well. Xolisile said,

Xenophobia in science exists. Black South African students suffer under the supervision of African foreign supervisors who intentionally frustrate them to the point that they leave and do not graduate. The African foreign students are praised by all races, and they graduate quickly. This is devious discrimination because Black students are afraid to complain or report this behaviour to the HoDs or Deans because they will be labelled as xenophobic and that is not true.

Nompumelelo’s and Xolisile’s experiences raise many issues. The first is that there is an insidious element occurring within certain science departments, where some African students are victimised because they are indigenous Africans. Nompumelelo narrates that the non-indigenous African foreigner is behaving in the same way some white people still behave but since it is another African person the only accusations they can levy are sexism, classism, and ethnicity, but it cannot be race. What is occurring here is overt ethnic discrimination and it perpetuates what the white, male, hegemonic science environment continues to claim, that African students, especially African women, are intellectually inferior. The second issue is that this situation is complicated because the African students are held hostage by how they complain about their mistreatment. They are afraid to complain about this non-indigenous African professor, because they will be labelled as xenophobic.

This further emphasises what was mentioned earlier in the paper that what is occurring in STEM disciplines is reform because the neoliberal and neo-colonialism that exist in the academy are so prevalent that when African students or staff challenge these ideologies they are labelled as ‘racist’ by the white people for wanting to dismantle the system. I ask the question: How do we resist temptation where those who are against decolonizing or those who support it only in rhetoric (mainly white academics) use non-indigenous African academics to block access to academia for the sole purpose of preserving the residual effects of colonialisation in South Africa? One could argue these non-indigenous African academics are also being used against the decolonisation project and other participants’ narrated experiences noted that some non-indigenous African academics in STEM were themselves perpetuating this blockage, now with a new purpose of reducing their vulnerability and securing themselves. This demonstrates how intersectionality operates in terms of competing for power and positions amongst marginalised and oppressed groups in higher education. The narratives by Xolisile and Nompumelelo expose the prevalent neoliberal and colour-blind ideology in STEM disciplines, which labels all individuals as either racist or xenophobic in order to silence and dismiss their experiences. This indicates that even if you remove white people in STEM and replace them with only African people (African foreigners included), the system of discrimination, sexism, ethnicity, and classism will continue in different ways and that once again emphasises that reform is occurring but not real transformation.

### ‘Transformation’ mutations

As previously mentioned, the STEM ontology values the Eurocentric characteristics of competition, individualism, survival of the fittest, and meritocracy (Herzig, 2004; Mayes-Tang, 2019). Institutional and departmental racism and sexism manifests and expresses itself in norms and values such as having a disproportionate number of white males in positions of authority. What happens then when certain individuals are deemed to be transformative, based on their race or gender, but their individual ideologies are not aligned with the transformation agenda. As Mbal’enhle mentions,

We have a research unit and there was a Black man who was elected as director and ever since he became director only white people have been hired for positions and he does not like Black people, yet he is Black. He is supposed to be the face of transformation, but he is setting the transformation agenda back by his actions.

Mbal’enhle, who is in Xx university, described how in her unit an African man was elected as a director and under his tenure only white men were hired for positions, and yet although he is in an authoritative position to enact sincere transformation, he consistently chooses not to do so. What does that mean in terms of furthering the transformation agenda. Literature has mentioned how STEM faculty members play an important role in co-constructing norms that mitigate racialised and gendered construction of stereotypes and making the STEM environment more inclusive (Featherstone et al., 2011). The oppression and overt exclusion of Africans especially African women within STEM confirm what Mboti (2021) has reiterated that apartheid continues to modify and is never the same thing. It continues to adapt and to hide more efficiently under certain ideologies such as colour-blindness and anti-blackness and it uses reform so it can be disguised as transformation when it is not. McGee and Bentley (2017) argue that Black within-group tensions exist in the west and their analyses of such tensions were based solely on race, leaving open questions about the interplay of racism with patriarchy. However, the examples of African women’s tensions with other African peers highlighted in this section were of instances of internalized racial-gendered oppression captured in the broader study, but because discovering this phenomenon was not the central focus of this current work.

The Department of Higher Education (DHET) in South Africa focuses on universities and institutions of higher education; the department of basic education solely focuses on education from pre-school to grade 12; the school boards and school leaders all have explicitly stated their mandate and goal, which is to increase the number of African students in science and mathematics disciplines. These entities of the DHET and the Department of Science and Innovation (DSI) have agendas and campaigns, such as the 2030 campaign which are strategic plans to recruit, train, and retain African women so that they will make up 50% of the teaching and research staff in the scarce skills of STEM (National Planning Commission, NPC 2012). What does that mean for achieving the 2030 agenda when participants are being actively prevented from pursing STEM degrees in universities in South Africa? Zandile mentioned her experience in mathematics and how she was intentionally prevented from beginning her mathematics degree by a Black administration. Zandile stated,

The reason why I started my Mathematics degree a year later than planned is because the department of education withheld my marks and would not release my Matric certificate. They said they had to investigate that my teacher did not give me the answers because no one has ever in [*] high school ever passed Matric higher-grade mathematics with an A. I think it is because they could not believe me, a poor township girl, from a township high school could get a distinction in Higher Grade^5^ Mathematics so they investigated and wasted a year of my life. I was discouraged because I have loved mathematics from a child and always done well and when I do something good it is unbelievable, yet now I am in my final year of PhD in mathematics.

Zandile’s narration highlighted how her experience of oppression and exclusion from mathematics begun in her secondary schooling, in Matric^6^ where she was able to achieve a distinction in mathematics from a Black township school which is severely under-resourced and understaffed and where African students historically have not succeeded or passed their high school sufficiently to enter universities. Zandile having overcome all the obstacles of being African and female and coming from a poorer socio-economic background was further victimized by a Black administration. This administration oppressed her by withholding her Matric results to validate her mathematics results forcing her to defer an entire year of university and to relinquish her scholarship for that year. These Black administrations are working against the transformation goals of the departments of higher education and basic education, by preventing an African child from achieving a university qualification in the scarce skills of STEM and therefore achieving social upward mobility and consequently breaking the cycle of poverty. There have been reports of strategic exclusion of African students choosing science and mathematics subjects in school, by reducing student numbers taking these subjects in township and rural schools in order to increase the pass rate. Consequently, these schools have an agenda to pass as many students as possible by discouraging students not to take mathematics or to take it on a lower level such as standard grade to increase their chances of passing mathematics. The implications of encouragement, gender, and school number of passes are all a result of pressure from the leadership of the school and department of education to meet certain targets and agendas at the expense of African students specifically African girls wanting to pursuing mathematics. This highlighted the Herculean efforts African women in STEM face and must overcome the enormous battles of racism, sexism, and classism that begins in high school and continues to university where they face the same persecutions from every race and gender as their advance and progress in South African universities. There appears to be a conflict in the STEM goal, because the government and leaders of education in South Africa want transformation in the STEM fields and have specific agendas and campaigns to increase the number of African girls and women in these disciplines, yet in the lower levels of high school, there appears to be a divergent agenda of actively excluding and discouraging African scholars from pursuing these subjects. Unless this conflict is resolved the number of African girls and women pursing STEM disciplines in South African universities will remain low.

The institutionalising of laws and behaviours in colonial and apartheid South Africa segregated the nation and discrimination and race was used as a structure to create division and as a marker of exclusion. The call for transformation was intended to redress this historical exclusion by including previously disadvantaged people in institutions of higher education. However, there needs to be a conversation around transformation that goes beyond demographic change only. There exists a belief that transformation has occurred in these fields due to the presence of white women who enjoy racial privilege and remain perpetrators of oppression themselves, as narratives by Fezile and other participants highlighted. There are some critics who demand that transformation can only occur when we replace white academics with African academics; that is not enough because what we will be doing is replacing one power (white men) with another (African men), as Nompumelelo, Thandeka, Fezile, and Mbal’enhle reiterated. The fact that African people are overrepresented in the lower ranks in the university structures and that the upper echelons are still dominated by white people is an example of the flaws in the transformation agenda. Due to the neoliberal structures of universities, white people still have the structural and institutional power and capabilities to prevent true transformation from occurring. Therefore, they cannot be excluded from the transformation agenda. White people as the internal hegemonic forces still have decision-making power to constantly ‘push back’ at transformational efforts; which begs the question, why do they remain at the top end of the hierarchy and African people remain in the lower ranks? This has and will continue to create new social hierarchies, which is what is occurring where issues such as ethnicity and citizenship are used as tools of exclusion in university structures (Idahosa, 2020). There will be no sustainability in the transformation agenda if the exclusion of white people and African foreigners continues to be reinforced. There needs to be a substantial reformed transformation agenda that moves beyond demographic transformation and closer to structural, institutional, and systematic transformation.

## Conclusion

The authors Battey and Leyva (2016) argue whiteness is the foundational concept for racism and I argue for all forms of oppression. Internalized racism is a consequence of white supremacy and white privilege which is the dual nature of privileging whites whilst simultaneously oppressing those outside the boundary of white. The actions of some African men and people on the transformation agenda deem representative of transformation in STEM indicate that white supremacy and Black inferiority have been internalized by some of them and are enacted on African women pursing STEM degrees. The internalized racism acts in the same way as white supremacy in which white supremacy in STEM education acts to reproduce subordination and advantage. Although STEM departments and institution proclaim neutrality and the colour-blind ideology, it is just a way whiteness as a construct continues to shifts over time, and continues to oppress those outside its boundary, namely African women. The oppression is through dialectical mechanisms: symbolic (ideologies) and material (resources) with a focus on the colour-blind ideology (Battey & Leyva, 2016).

Even though African women in STEM have made inroads into white dominated fields, they continue to face epistemic injustice, epistemic marginalization, presumed incompetence, intellectual inferiority, and the cognitive dissonance of consciously recognizing the white supremacy that pervades the STEM culture as the participants in this study have reiterated. While African men in STEM face racism-related epistemic challenges, African women in particular are in a double bind, subject to both racism and sexism. The African women must endure continually racial battle fatigue, which is bought on by racial-related stressors (McGee, 2021, p. 38). This type of fatigue can cause debilitating psychological and physiological stresses. African women are doubly compounded with the stress of sexism as well. Some participants reiterated that they just wanted to finish their doctorate and leave academia. These are elements that policy makers and STEM departments need to be aware of if they want to transform STEM academia. An additional stressor that has occurred for some of the participants is an inability to be assisted when they encounter discrimination specifically pertaining to them as indigenous African women students. This exposes the violence of white supremacy, in collaboration with African non-indigenous people, inflicting and perpetuating the ideology of indigenous African students being unintelligent, lazy, and undeserving of all the good things their government gives them (funding for higher education). There is also the problem of having African university administrators who are prevented from acting against the discrimination and psychological violence the students face because of the repercussions that would follow by acting in such a way that appears to be Afrophobic and xenophobic in nature.

In order for authentic transformation to occur and not just demographic reform, we need to improve STEM disciplines and culture and perhaps improve the quality of African women’s experiences. Universities must enact policies to change the institutional culture. The measures they could take could include implementing a new policy by appointing African women into positions of authority and by building support structures such as mentoring young African women students in STEM. Another salient factor is the fact that in order for institutional culture to change significantly, it must not just be the African students who have to change, adapt, and assimilate into the STEM environment, but academics and administrators need to change too. There needs to be transformation in important authoritative positions because allowing white men to remain in these positions can strategically consciously and unconsciously encourage racism. This is how racism and sexism become institutionalised because certain individuals have the professional power to continue to enact it.

There are many ways we can genuinely transform STEM disciplines. For example, we can change the way science is taught, and decolonize it by instituting African solutions to solve African problems. We could make it more human and less competitive and cutthroat. Supervisors need to be trained on how to supervise various types of students and exercise better social skills, as well as pursuing the removal of the neoliberal ideology of meritocracy because South African students are not equal in their socio-economic or secondary schooling backgrounds. The majority of African students come from poorer socio-economic backgrounds and disadvantaged secondary schooling compared to their white counterparts. If genuine equality is to prevail, then STEM disciplines need to level the playing field for all. Fundamental change and transformation are needed in higher education and institutions. However, decolonisation and transformation largely remain buzzwords, with a focus on performative change and *window dressing*. There are ways in which STEM can move from the demographic reform that is occurring right now, towards transformation, when they stop purporting that reform is transformation when it is not.

## Footnotes

1 Afrikaans is a language of southern Africa, derived from Dutch which was brought to the Cape by settlers in the seventeenth century. There is a distinction between historically white and historically white Afrikaans universities. The historical white institutions were English speaking and are heavily influenced by the British colonisers of South Africa. The historically white Afrikaans universities were of the Afrikaner nation which was derived from the Dutch settler in South Africa.

2 Afrikaans is a language of southern Africa, derived from Dutch which was brought to the Cape by settlers in the seventeenth century. There is a distinction between historically white and historically white Afrikaans universities. The historical white institutions were English speaking and are heavily influenced by the British colonisers of South Africa. The historically white Afrikaans universities were of the Afrikaner nation which was derived from the Dutch settler in South Africa.

3 In South Africa, races were classified as Black and within that classified further categorised into groups. African meant African people indigenous to South Africa, Coloured referred to people who were of mixed race, and Indian were people who came from India. Even within the category ‘Black’ historically the Indians and Coloureds were more privileged in terms of access to resources and education and the Africans were not.

4 I refer to African students as still disadvantaged and not ‘previously’ disadvantaged. Even though African students have access to previously denied areas of study, they remain disadvantaged due to their unequal secondary schooling and lack of preparation for higher education.

5 In South Africa, certain subjects like mathematics are offered in two levels: higher grade mathematics and standard grade mathematics (mathematics literacy).

6 Grade 12 in South Africa is commonly referred to as Matric. Short for matriculating your final year in high school.
